# Bodyweight at Birth and Growth Rate during the Neonatal Period in Three Canine Breeds

**DOI:** 10.3390/ani10010008

**Published:** 2019-12-19

**Authors:** Magdalena Schrank, Antonio Mollo, Barbara Contiero, Stefano Romagnoli

**Affiliations:** Department of Animal Medicine, Production and Health, University of Padua, 35020 Padua, Italy; antonio.mollo@unipd.it (A.M.); barbara.contiero@unipd.it (B.C.); stefano.romagnoli@unipd.it (S.R.)

**Keywords:** puppy, birthweight, neonatal growth, litter size, daily gain

## Abstract

**Simple Summary:**

The 349 recognized canine breeds differ greatly in bodyweight and, therefore, in birthweight and neonatal growth. The weight and growth of puppies are easily measurable, and are possible early indicators of problems. Low birthweight has been linked to neonatal mortality based on results obtained by grouping breeds according to their adult bodyweight. Breed-specific ranges of birthweight and growth would allow for the identification of puppies at risk. Our aim was to evaluate the birthweight and early growth of healthy puppies of three breeds in a breed-specific manner. Birthweight, expressed as percentage of mothers’ bodyweight, showed that puppies of a large breed are born smaller than puppies of a small breed. Puppies of a large breed gain weight slower than puppies of a small breed. Sex has no impact on birthweight, whereas litter size influences birthweight and weight gain. Based on our procedure, we considered 29 of 213 puppies to be of a low birthweight, whereas 160 of 213 might have been considered of a low birthweight if using the classical criteria (based on breed groups). This shows the importance of breed-specific evaluations of birthweight. Further research is needed on the importance of breed-specific evaluations for early growth.

**Abstract:**

Weight at birth (bBW) and early weight gain have been linked to the risk of neonatal mortality. Pups are described to be of low bBW if weighing less than one standard deviation (SD) below the mean. Most studies classified breeds according to their expected adult bodyweight. Our aim was to evaluate the breed specificity of these parameters. We assessed the bBW of 213 puppies of Bernese Mountain Dog (BMD), Tibetan Terrier (TT), and Lhasa Apso (LA) breeds, as well as the neonatal growth rate of 133 puppies of BMD and TT. BMD puppies were born relatively smaller than puppies of TT and LA (*p* ≤ 0.0001) and gained less weight than TT puppies during the first 14 days (*p* ≤ 0.05). Litter size had a significant impact on bBW and daily gain until the onset of the third week for BMD (*p* < 0.0001 and *p* = 0.0005, respectively) and TT (*p* = 0.0003 and *p* = 0.0064, respectively). When using bBW means and SD specifically assessed according to breed, 29 out of the 213 neonates of our study were judged as being of low bBW, whereas, when using the classical criteria (based on breed groups), the number of low bBW pups was 160 of 213. These results suggest that evaluations of bBW and neonatal growth should be performed in a breed-specific manner.

## 1. Introduction

The domestic dog represents a species with a wide variability in phenotypical appearance due to the abundance of its breeds. The Fédération Cynologique Internationale (FCI) recognizes 349 breeds, ranging from Chihuahuas with a minimum adult bodyweight of 500 g to breeds weighing over 100 kg, such as Spanish Mastiffs and Saint Bernards [1]. Neonatal mortality in domestic dogs is high, and it is therefore of great interest for breeders to find parameters which may permit the identification of puppies at risk of neonatal mortality. Dog breeders, both hobbyists and professionals, are continuously trying to improve, and are therefore searching for easily applicable methods to improve neonatal survival. Although, for decades, responsible breeders have been using weight to estimate the wellbeing of puppies and mothers, and experienced breeders may have an estimation of when a puppy of a respective breed is of low bodyweight, no breed-specific charts exist. Therefore, knowledge of physiological birth bodyweight and the neonatal growth curve of the respective breed would be useful for the breeder and the treating veterinarian. Weight is an easily measurable parameter and, confronted with other parameters which might give information on the wellbeing of a neonate, less error-prone. During the daily process of weighing, breeders may further identify particularities which are not as easily visible when observing the puppies in the group. Moreover, for the individual puppy, early contact with humans may have beneficial future effects. The weight curves and growth rate of dogs have been investigated in several studies [2,3,4]. Weight has an impact not only on health [5] but also on certain behavioral aspects, with larger female puppies being more active and explorative than their smaller counterparts [6]. The growth of puppies after weaning age has been investigated [7,8]. However, little is known about breed differences in birthweight and normal growth patterns within the neonatal phase—the first three weeks of life [9,10]—a very delicate phase of life during which puppies are highly susceptible to health problems. Canine neonatal mortality is estimated to account for 20% of all puppies born until the age of 7–8 weeks, with 75%–90% of deaths occurring within the first three weeks of life [9,11,12,13]. Correlations between low birth bodyweight (bBW), growth rate within the first 2–4 days of life and neonatal mortality in the canine species have been reported [9,12,14,15,16] for groups of breeds. Previously published studies on canine bBW used a population comprising puppies of different breeds grouped according to their expected adult bodyweight. To date, there are no rules on how to group dog breeds according to their bodyweight, which leads to important differences in the composition of the populations of these studies. For instance, the Tibetan Terrier has been considered as a dog of small size [16] or of medium size [12], depending on the weight ranges used to describe breeds of either small or medium size. However, no breed-specific bBW cutoff value has yet been established above which a canine neonate is to be considered normal. We included, in our investigated population, puppies of large, medium, and small breeds to permit comparison with previously published results and to show the differences in birthweight and growth. The aims of the present study were to investigate (a) ranges of physiological bBW specific for Bernese Mountain Dogs (BMD), Tibetan Terrier (TT), and Lhasa Apso (LA) breeds; (b) ranges of physiological daily weight gain specific for puppies of the BMD and TT breeds; and (c) possible influencing factors, such as breed, sex, birth sequence and litter size, on birth bodyweight and neonatal growth.

## 2. Materials and Methods

The breeds included in the present investigation are BMD, TT, and LA. The respective FCI standards describe the BMD as a breed above medium size [17], TT as a breed of medium size [18] and the LA as small-sized breed [19]. A total of 213 puppies from 38 litters of the above three breeds born in two different kennels (BMD and TT in Kennel A and LA in Kennel B) between 1994 and 2018 were included in the statistical analysis (Table 1). Seven puppies of BMD and TT were either stillborn (n = 5) or died during the neonatal period (one due to diffuse hemorrhage on Day 6 and one due to trauma on Day 16) (Table 1), and were excluded from statistical analysis. Information on birth body weight and daily growth within the first 21 days of life was available for a total of 133 puppies of BMD and TT born from Kennel A, while, for 80 LA puppies born within the same period of time from Kennel B, information on birth body weight was available (Table 1). Collected information about bitches included breed, date of birth, parity, and non-pregnant bodyweight. Information available about puppies included breed, date and hour of birth, birth order, sex, and bBW. Birth order describes the position of the puppy in the birth sequence. The day of parturition was considered as Day 0 and the weight on Day 0 was considered the bBW. All puppies of BMD and TT were weighed at least once daily for the first 21 days of life. In the case of a dystocia, documentation was provided about when during parturition, and which puppies were delivered via an emergency caesarean section.

Information about the health of puppies and the dam was available and taken into consideration to rule out any maternal condition which could endanger neonatal health.

Birth bodyweight of puppies was evaluated in grams (g) and as a percentage of non-pregnant mothers’ bodyweight. Values in g are referred to as absolute bBW and values in percentage are referred to as relative bBW. The total litter weight was calculated as the sum of the bBW of puppies of each litter and reported as the percentage of the mothers’ bodyweight prior to mating.

The average daily gain of neonatal weight is calculated as the difference between the day of interest and the previous day. The average daily gain is given as the gain in g/day (ADGg) or as percentage of the birth bodyweight (ADG%). Multiplicity describes the time (days) needed for a puppy to double and triple its bBW. Therefore, multiplicity is calculated as the difference between the day of interest and the day of birth.

Dams were weighed prior to mating. Puppies were weighed after colostrum intake on the day of birth. Thereafter, weighing was performed by the breeder at the same time each day. A total of three different kitchen scales were used over the study period. In Kennel A, the scales used were a Tupperware Digital Scale with a range from 1 g to 3 kg (Tupperware, Orlando, FL, USA) and a Soehnle Page Profi digital scale with a range from 1 g to 15 kg. In Kennel B, the scale used was the Zenith Skala analogic scale with a measurable maximum of 26 kg (Zenith, West-Germany, Germany).

### Statistical Analysis

Descriptive statistics were performed using Microsoft Excel for Mac 2016, Version 16.16.4. All analyses were performed using SAS (9.4 release, SAS Institute Inc., Cary, NC, USA). Separate analyses by breed were conducted for bBW using a split-plot model with two levels of error. The linear ANOVA model included in the main plot the fixed effects of mother, parity, litter size (three, four, five, six, seven, eight, nine, and more than nine puppies) and litter effect as main error line; in the second plot, the gender of puppies and order in the birth sequence. To allow comparisons among breeds, puppies’ bBW and the sum of the bBWs of each litter (expressed as percentage of the bodyweight of their non-pregnant mother) and the average daily gain (ADG%, expressed as percentage of the bBW) were analyzed using the same ANOVA model, which also included the fixed effect of breed. For ADG, separate analyses were conducted for each week of growth. Doubling and tripling of weight were analyzed in the same way. Post hoc multiple comparisons among levels were performed using the Bonferroni correction. A factor was considered to have a significant impact if *p* < 0.05. The impact of the individual mother and the individual litter is referred to as the “mother-effect” and the “litter-effect”.

## 3. Results

The results are presented in three sections. Section 3.1. (general evaluation and bBW) includes a description of the litters, litter size, and the bBW of the three breeds. bBWs are initially given in g and are then presented as percentage of their mothers’ bodyweight to allow comparison among breeds. Section 3.2. (average daily gain (g)) features the growth of BMD and TT puppies during the first 21 days of life presented in a descriptive manner. Section 3.3. (average daily gain (%) and multiplicity) reports the daily gain as percentage of the bBW of BMD and TT puppies to allow comparisons between the two breeds and multiplicity.

### 3.1. General Evaluations and Birth Bodyweight

The range of litter sizes, mean litter sizes, ranges of mean bBW (g), and ranges of individual bBWs (g) are presented in Table 2. Litter size: the ranges of litter size were 4–11, 3–8, and 3–7 in the BMD, TT, and LA litters, respectively. Neither the age of the dam nor parity had a significant influence on litter size. However, the breed of the dam had a significant influence (*p* < 0.05). Birth bodyweight (in g): the absolute bBW was evaluated separately for each breed and the *p*-values of possible influencing factors are given in Table 3. Puppies of larger litters were born significantly smaller than puppies of smaller litters in BMD and TT. The mother-effect was statistically significant in BMD and TT litters, and the litter-effect was statistically significant in TT and LA (Table 3). bBW (in %): comparison of the bBW in terms of percentage of the mothers’ bodyweight showed that BMD puppies were born smaller than TT and LA puppies (*p* < 0.0001). Puppies of BMD weighed 1.3% of mothers body weight. The bBW (%) was 2.5% and 2.1% for LA and TT, respectively (*p* = 0.5). Sex had no impact on bBW (%). Total litter weight: the sum of the bBWs of all puppies of each litter expressed as percentage of their mothers’ bodyweight were compared among breeds. The average weight of a BMD litter was 9.6% of their mothers’ bodyweight and, therefore, significantly lighter than the weight of LA (*p* = 0.0022) and TT (*p* = 0.0008) litters, which weighed 11.6% and 11.9% of their mothers’ bodyweight, respectively. The size of the litter (*p* < 0.0001) and the breed (*p* = 0.001) had a significant impact on the total litter weight.

### 3.2. Average Daily Gain (g)

No weight loss within the first 48 h was observed in either breed. The ADGg for the BMD puppies ranged from 60.8 ± 1.4 g/day to 89.6 ± 2.4 g/day and was significantly influenced by litter-effect (*p* < 0.0001) and sex (*p* = 0.0202). When evaluating the influence of sex, the results show that female puppies gained significantly more weight (g/day) than males (*p* = 0.0202). The ADGg of the TT puppies ranged from 29.9 ± 1.2 g/day to a maximum of 47.0 ±1.50 g/day and was significantly influenced by sex (*p* = 0.0112) and bBW (*p* < 0.0001). In contrast to BMD, TT male puppies gained more weight than females. Parity and birth order had no influence on average daily gain in both BMD and TT puppies.

### 3.3. Average Daily Gain (%) and Multiplicity

**Figure 1:**
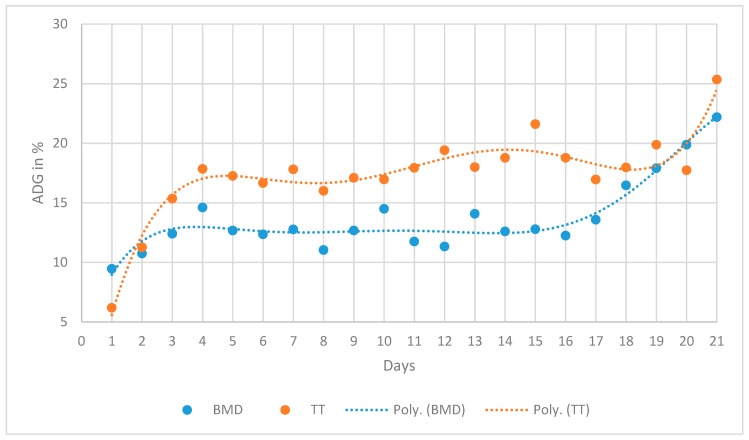
Average daily gain in percent (ADG%) for BMD and TT puppies over the neonatal period; BMD = Bernese Mountain Dog; TT = Tibetan Terrier; poly. = graphical polynomial interpolation of the data.

Puppies of BMD gained significantly less during the first and second week than puppies of TT. This changed in the third week, yet, with the increase in the ADG% of BMD, the difference lost statistical significance (Table 4 and Table 5). Breed and litter size had a significant impact; however, significance was not continuously expressed on each single day of the first and second week. Litter size had a significant impact on ADG% during the first and second week, with puppies of large litters gaining less than puppies of smaller litters (Table 5). Sex had no significant impact on ADG% within a breed, yet, when comparing BMD males with TT males, a significant difference in daily gain could be observed. TT males gained significantly more than BMD males in the first and second week (Table 4). Changes in ADG% are illustrated in Figure 1. When evaluating multiplicity, breed showed a continuous, significant impact from Day 7 onwards (*p* < 0.05). BMD puppies doubled their bBW in 10 ± 0.3 days, which is significantly later than puppies of TT (8.7 ± 0.2 days) (*p* = 0.0004). Litter size had no impact on the multiplicity of bBW, whereas the sex of the puppy did: BMD males needed significantly more time (10.6 ± 0.4 days) to double their weight than BMD females (*p* = 0.0486), TT males (*p* = 0.0004), and TT females (*p* = 0.0023); in every comparison, TT males were the fastest (8.5 ± 0.2 days) in doubling their weight. BMD puppies were slower in tripling their bBW than TT puppies (17.9 ± 0.4 days vs. 14.3 ± 0.3; *p* < 0.0001). The impact of sex between breeds was also statistically significant with regard to tripling. BMD males needed significantly more time (18.6 ± 0.6 days) to triple their weight than TT males and females (*p* < 0.0001) (Table 6).

## 4. Discussion

### 4.1. Limitations

The total population size of our study (213) may be perceived as a limitation of the present study when compared to the total number of pups from other studies, such as 2373 [12], 789 [20], or 3292 [21]. However, we focused on only three breeds and, in fact, the number of puppies per breed does not differ considerably. In the present study, 89 puppies of TT, 44 puppies of BMD, and 80 puppies of LA were included. In the abovementioned studies, the number of puppies of BMD was 88 [20] and 196 [21], the number of TT puppies was 2 [21], and the number of LA puppies was 5 [12].

The interval of time between the first and the last litter born in our population may be considered as another limitation, as international breed standards and/or breeding techniques may change with time. Selecting for larger or smaller adult size in certain breeds may have an impact on bBW. Fourteen years is probably a long enough period to obtain significant changes in size and the bodyweight of adult dogs, which may impact bBW. However, the international standards of the three breeds of our study did not change over the last three decades with regard to height and bodyweight. During the 14 years of the study, the three breeders continued to use the same criteria with regard to achieving a certain phenotype by selection [22]. Nutrition of the dam, as well as the housing and handling of both the dam and the puppies, remained unchanged at the kennels during our study. The balances were changed once in Kennel A, whereas in Kennel B the breeder used the same balance throughout the studied period. The influence of the change of balance on the data in Kennel A is difficult to understand in a retrospective manner. We consider the impact as marginal, as the individual birth body weights between litters of similar size weighed with different balances did not differ importantly. Nevertheless, future research and data collection in this field should be obtained using one balance, therefore standardizing data collection to avoid any kind of bias. Over time, slight changes may have been observable in the phenotypical aspects of each of the three investigated breeds. This may be attributed to individual preferences of breeders in the selection of the dam and the sire. Breeders may therefore select animals nearer to the lower or upper range of the described size in the FCI standard of each respective breed. In this context, we consider the impact of time to be of no importance for the population of the present study as throughout the years breeders maintained the same ideas regarding the phenotype they wanted to achieve by selection. For the future we would consider a breed-specific evaluation of birth body weight and growth as useful, including different breeders for each breed. In this way, any possible influence of personal selection would be decreased to a minimum. We think that complete exclusion of selection-dependent influence on the phenotype of any breed is impossible, as selection and therefore phenotype may differ not only between breeders and changes over time, but also between regions and countries.

### 4.2. Litter Size

Mean litter sizes are reported to vary widely for the breeds investigated in our study [10,23,24]. The results of the present analysis, as well as the findings of previous reports, are compared in Table 7. The mean litter size of TT in our population is larger than those reported in the literature. Similar results could be found for the BMD breed, as only Groppetti et al. [20] reported a larger litter size. The larger variety in reported litter sizes for BMD may be due to the fact that, in large breeds, the litter size is mainly dependent on the prolificacy of the dam, whereas in smaller breeds, the litter size is further limited by the size of the abdomen. Groppetti et al. [20] described litter size as being influenced by maternal size, weight, age, and parity. In our studied population, age and parity had no significant influence on litter size, whereas breed, and therefore size/weight, had a significant influence (*p* < 0.05).

### 4.3. Birth Bodyweight

The breed-specific description of bBW for our studied breeds is not uniform, as different authors have reported either the mean, the median, or a range. For BMD puppies, the mean and median bBW has been reported to be 588.9 ± 88.4 g [20] and 541 g, respectively (range 490–600 g) [21]. For puppies of TT, the median bBW has been reported to be 217 g with a range of 215.5–218.5 g [21], whereas for LA puppies, the mean of bBW has been reported to be 242 g with a range of 228–256 g [12]. Our observed means for BMD and LA puppies, and the medians for BMD and TT puppies, are in line with those already reported [12,20,21]. Birth bodyweight has further been evaluated on the basis of different breeds grouped according to their expected adult bodyweight. In these cases, the bBWs of BMD puppies of the present study are similar to the mean and median bBW of puppies of the group of large-sized breeds reported in previous studies [15,16,20]. The importance of breed-specific evaluation becomes evident when comparing our results with the results reported by Gill [12] and Mila et al. [16]. Grouping breeds differently resulted in the TT being either a dog of small size [16] or of a medium size [12]. The bBWs of many of our litters differed from previously reported means and were found to be both above and below [16,20]. The reason for the lack of agreement between Mila [16] and Gill with regard to the lower threshold of bBW may be due to the different compositions of groups of breeds in their studies. Our results are in agreement with those reported by Mila et al. [16], while they are in complete contrast with the findings of Gill [12], who considers puppies’ bBW as low if it is lower than one standard deviation below the mean [25]. If we apply this method to our results, eight BMD, 87 TT, and 65 LA puppies should be considered underweight, and therefore at risk of neonatal mortality. In addition, assuming the exclusion of TT and LA puppies of litters which were born with a bBW of 100–130 g, and therefore on the lower extreme of possible bBW, the remaining 84 and 61 puppies of TT and LA breeds, respectively, would nevertheless be considered underweight, and therefore susceptible to complications and death within the first few days of life [12,16]. When applying the rule using means and standard deviation calculated on the basis of our breed-specific population, only seven BMD, 13 TT, and nine LA puppies weighed less than one standard deviation below the mean. This important difference may show that the application of the method of Grünwald [25] may not be useful if the means and the standard deviation are calculated for breed groups rather than a specific breed, and demonstrates the importance of breed-specific evaluations of this parameter. Therefore, when evaluating the physiological bBW of a specific breed, the calculation of a cutoff value of bBW may be biased if breeds are grouped according to their expected adult bodyweight rather than being evaluated separately. A lack of consistency in possible influencing factors of bBW in the breeds of our study has already been reported [16,20,21,26,27]. We observed a significant effect on bBW of breed (for all three breeds) and of litter size for BMD and TT litters. We did not observe a significant effect of gestational length and maternal factors such as weight, age, size, and breed, unlike what was reported by others [20]. Reports of the significant impact of sex on bBW are inconsistent. A significant sex dimorphism was described by some authors, with males being born significantly heavier than females [21,26,27], whereas other studies found no significant impact of sex on the bBW [16]. Moreover, no significant effect of sex dimorphism on bBW was observed in the present study. The litter-effect is to be considered a complex sum of factors influencing all puppies of the same litter, yet not all litters of the same mother. No clear definition for this effect is currently available, although genetics and environment may well play a role. In our study, the litter-effect had a significant impact on bBW only in TT and LA litters. A significant litter-effect has been reported, yet, due to the study design, it is unclear whether this effect may be present in all breeds [16,27]. Lawler [13] includes the litter-effect when concluding in his study that bBW is influenced by parental age, state of health, placental sufficiency, litter size, gestational nutrition, infections, and environment. Such a conclusion cannot be supported from our results. Further research is needed to clarify the mechanisms behind the litter- or the mother-effect. A negative correlation in comparison of weight ratio between weight of the mother and bBW of the puppies has been previously reported [1] and is supported by the findings of the present study. Puppies of a large breed are therefore born smaller than puppies of a medium or small breed when comparing the bBW in terms of percentage of mothers’ body weight. When evaluating the bBW as a percentage of the mothers’ bodyweight, the puppies of BMD weighed significantly less than TT and LA puppies. Fiszdon et al. reported that the sum of the puppies’ bBWs of a litter should not exceed 15% of the mothers’ bodyweight [1]. In the present study, five litters (three TT and two LA) had a weight of over 15% of the mothers’ bodyweight, with the heaviest litter weighing 18.4%.

### 4.4. Neonatal Growth

Comparing our results with the past literature is somewhat difficult, as most studies looking at canine growth rate during the first three weeks of life used a population consisting of different breeds [1,14,15,27]. During the first and second week of life, breed, litter size, and sex, divided by breed, had a significant effect on ADG%. All the factors lose their significance from Day 15 onwards. Mila et al. [15] evaluated growth within the first two days of life (considering it as early growth) and reported a mean growth rate of 3.3% for puppies alive at Day 2, ranging from −4.9% to 13.2%, calculated for a population composed of various breeds of different sizes. The reported early growth rate of puppies still alive at Day 21 (5.1% with a range of −2.2% to 13.2%) [15] is closer to our findings. However, an important difference remains, with puppies of the present population greatly exceeding the ADG% reported by these authors. Mila et al. [15] reported that the early growth rate is predictive of survival within the first 21 days, and proposed a cutoff value of −4%, under which puppies are more likely to die than puppies which lose less weight or gain weight. Such a threshold could not be tested in our study due to the lack of significant neonatal mortality and the difference in the study design. Our results, which show a small breed gaining more weight each day and growing faster than a large breed, support the findings of Fiszdon et al. [1]. Our results are in agreement with Indrebø et al. [9], who reported an average growth of 8% within the first three days and 12% in the following four days. Furthermore, Hoskins [28] suggested that a daily weight gain of 10% (with an increase of 2–4 g/kg (adult BW)/d during the first five months of life) should be considered desirable. The puppies in the present investigation maintained a weight gain of >10% from Day 2 onwards. Both breeders and veterinarians consider a weight loss of new-born puppies within the first 48 h as normal, yet the reports are inconsistent. Different authors, e.g., Mila and co-authors [14,15] and Bigliardi et al. [26], reported that an initial weight loss during the first 2–4 days of life is not uncommon, and may have a negative impact on neonatal survival. Indrebø et al. [9] considered a weight loss of up to 5% within the first 24 h possible, similarly to Bigliardi and co-authors [26], who described an average weight loss of 11.3 ± 2.3 g (corresponding to 2.3%) in Boxer puppies within the first three days, with larger puppies showing a smaller weight loss. Nevertheless, Bigliardi et al. [26] proposed that the weight loss should in no case exceed 10%, and Münnich [29] advised supplemental feeding if the weight loss in the first 24 h of life is more than 10% of their birthweight. Mila et al. [14] concluded that puppies which lose weight between birth and Day 4 are at a higher risk of death within the first three weeks of life compared to littermates which gain weight during the same period. On the contrary, Hoskins [28] stated that healthy puppies should gain weight continuously. No weight loss could be observed in our studied population. Breeders consider the multiplicity of weight an important indicator of the wellbeing of their puppies and the milk yield and nursing capacity of the mother. Puppies of TT and BMD of the presently investigated population doubled their weight within 8.7 ± 0.2 days and 10.0 ± 0.3 days, respectively. Breed, and sex divided by breed, had a significant impact on the time needed to double weight, with BMD males needing significantly longer than BMD females, TT males and TT females. Overall, TT males were the fastest, with reports of the time needed to achieve doubling of weight ranging from seven to 14 days [1,9,26,30,31]. Considering these reports and the findings of the present study, the perception of breeders that puppies should double their weight around day 10 may be considered realistic.

### 4.5. General Considerations and Implications for Future Research

Our results, although obtained from a relatively small number of puppies, show the necessity of further research. The evaluation of a large number of puppies and the interpretation of results using breed groups could seem a time- and resource-saving approach to the subject of birth bodyweight and its implications for the breeder and the puppy. Our results, especially considering the application of the rule of Grünwald [25], show how large the difference between a breed-specific and a breed-group based approach is. Experienced breeders may have an estimated cutoff value above which they consider their puppies normal, yet there is no chart which may be used by less experienced breeders or veterinarians who may be confronted with a high number of different breeds. For both inexperienced breeders and veterinarians, such a chart might be useful, as it would permit the appropriate identification of puppies of a low birth bodyweight and increase their probability of survival by receiving special attention, such as additional feeding or complete hand rearing.

## 5. Conclusions

Comparing the results of different studies on puppies’ bBW and their growth rate during the neonatal period is difficult, due to the differences in the composition of the population. There is no generally accepted rule on how to create breed classes on the basis of adult bodyweight. Considering the variety of dog breeds, a breed-specific evaluation of normal bBW and neonatal growth rate is warranted before attempting to create cutoff values below which puppies should be considered at risk. Differences between the results of the present and previous studies show the importance of breed-specific evaluation of these parameters.

## Figures and Tables

**Table 1:** General information on the population.

	Kennel	N Dams	N Litters	N Puppies (Alive/Dead)
BMD	A	3	6	44/5
TT	A	6	16	89/2
LA	B	6	16	80/0

BMD = Bernese Mountain Dog; TT = Tibetan Terrier; LA = Lhasa Apso; N = number.

**Table 2:** General information on litters and birth bodyweight (bBW).

	Litter Size (Range)	Litter Size (Mean ± SD)	Range Mean bBW ± SD (g)	Range Individual bBW (g)
BMD ^a^	4–11	7.3 ± 2.5	462 (±62)–662 (±26)	380–720
TT ^a^	3–8	5.6 ± 1.4	150 (±9)–334 (±14)	100–356
LA ^b^	3–7	5.0 ± 1.2	137 (±8)–230 (±7)	100–260

BMD = Bernese Mountain Dog; TT = Tibetan Terrier; LA = Lhasa Apso; N = number; range mean bBW (g) = range of mean bBW of all litters; range individual bBW (g) = range of bBW recorded for individual puppies of all litters of each breed; breeds with the same superscript gave birth in the same kennel.

**Table 3:** Significance of factors influencing the birth bodyweight.

	Litter Size	Mother-Effect	Litter-Effect	Sex of Puppy	Birth Order
BMD	*p* < 0.0001	*p* = 0.012	n.s.	n.s.	n.s.
TT	*p* = 0.0003	*p* = 0.0195	*p* = 0.0019	n.s.	n.s.
LA	n.s.	n.s.	*p* < 0.0001	n.s.	n.s.

BMD = Bernese Mountain Dog; TT = Tibetan Terrier; LA = Lhasa Apso; n.s. = not significant.

**Table 4:** Average daily gain in percentage of the bBW (ADG%).

	N Puppies	1st Week	2nd Week	3rd Week
BMD	44	12.1 ± 0.6 ^A^	12.99 ± 1.0 ^B^	17.99 ± 1.5
TT	89	14.66 ± 0.4 ^A^	16.7 ± 0.7 ^B^	14.31 ± 1.0

BMD = Bernese Mountain Dog; TT = Tibetan Terrier; N = number; values with the same superscript are significantly different (*p* < 0.05), ^A^: *p* = 0.0005; ^B^: *p* = 0.0064.

**Table 5:** *p*-values of factors influencing the ADG %.

	Breed	Litter Size	Sex	Breed Males (13 BMD vs. 43 TT)	Breed Females (31 BMD vs. 46 TT)
1st week	0.0005	0.001	n.s.	0.0033	n.s.
2nd week	0.0064	<0.0001	n.s.	0.0159	n.s.
3rd week	n.s.	n.s.	n.s.	n.s.	n.s.

BMD = Bernese Mountain Dog; TT = Tibetan Terrier; breed males = BMD males vs. TT males; breed females = BMD females vs. TT females; n.s. = not significant; significant = *p* < 0.05.

**Table 6:** Multiplicity of birth bodyweight (bBW) and its influencing factors.

	Breed (Mean ± SD)		BMD Males vs. Females (Mean ± SD)		TT Males vs. Females (Mean ± SD)	
Double	BMD: 10 ± 0.3 d TT: 8.7 ± 0.2 d	*p* = 0.0004	Males: 10.6 ± 0.4 d Females: 9.5 ± 0.3 d	*p* = 0.0486	Males: 8.5 ± 0.2 d Females: 8.8 ± 0.2 d	*p* = 1
Triple	BMD: 18 ± 0.4 d TT: 14.3 ± 0.3 d	*p* < 0.0001	Males: 18.6 ± 0.6 d Females: 17.3 ± 0.4 d	*p* = 0.1993	Males: 14 ± 0.3 d Females: 14.7 ± 0.3 d	*p* = 0.4356

BMD = Bernese Mountain Dog; TT = Tibetan Terrier; d = days, significant = *p* < 0.05.

**Table 7:** Mean/median litter sizes reported in the current and previous studies.

	Present Study	Borge et al. 2011 [23]	Tønnessen et al. 2012 [10]	Leroy et al. 2015 [24]	Groppetti et al. 2015 [20]	Groppetti et al. 2017 [21]
BMD	7.3 ± 2.6 ^c^	6.4 ± 0.3 ^c^	5.7 ± 0.2 ^a^	5.5 ± 2.8 ^c^	7.8 ± 3.2 ^c^	6 ^b^
TT	5.6 ±1.4 ^c^	5.2 ± 0.3 ^c^	5.1 ± 0.3 ^a^	n.g.	n.g.	2 ^b^
LA	5.0 ± 1.2 ^c^	4.9 ± 0.3 ^c^	4.7 ± 0.3 ^a^	n.g.	n.g.	n.g.

BMD = Bernese Mountain Dog; TT = Tibetan Terrier; LA = Lhasa Apso; n.g. = not given; ^a^ = litter size at Day 8; ^b^ = median litter size; ^c^ = mean litter size.
